# Difference in pulmonary permeability between indirect and direct acute respiratory distress syndrome assessed by the transpulmonary thermodilution technique: a prospective, observational, multi-institutional study

**DOI:** 10.1186/2052-0492-2-24

**Published:** 2014-03-25

**Authors:** Kenichiro Morisawa, Shigeki Fujitani, Yasuhiko Taira, Shigeki Kushimoto, Yasuhide Kitazawa, Kazuo Okuchi, Hiroyasu Ishikura, Teruo Sakamoto, Takashi Tagami, Junko Yamaguchi, Manabu Sugita, Yoichi Kase, Takashi Kanemura, Hiroyuki Takahashi, Yuuichi Kuroki, Hiroo Izumino, Hiroshi Rinka, Ryutarou Seo, Makoto Takatori, Tadashi Kaneko, Toshiaki Nakamura, Takayuki Irahara, Nobuyuki Saitou, Akihiro Watanabe, PiCCO Pulmonary Edema Study Group

**Affiliations:** Department of Emergency and Critical Care Medicine, St. Marianna University School of Medicine, 2-16-1, Sugao, Miyamae-ku, Kawasaki-shi, Kanagawa-ken, 216-8511 Japan; Division of Emergency Medicine, Tohoku University Graduate School of Medicine, Sendai, Miyagi, 980-8574 Japan; Critical Care Medical Center, Kinki University Hospital, Osakasayama, Osaka, 589-8511 Japan; Advanced Emergency and Critical Care Center, Nara Medical University Hospital, Kashihara, Nara, 634-8521 Japan; Department of Emergency and Critical Care Medicine, Fukuoka University Hospital, Fukuoka, Fukuoka, 814-0180 Japan; Advanced Emergency and Critical Care Center, Kurume University Hospital, Kurume, Fukuoka, 830-0011 Japan; Department of Emergency and Critical Care Medicine, Aidu Chuo Hospital, Aiduwakamatsu, Fukushima, 965-8611 Japan; Department of Emergency and Critical Care Medicine, Nihon University School of Medicine Itabashi Hospital, Itabashi, Tokyo, 173-8610 Japan; Department of Emergency and Critical Care Medicine, Juntendo University Nerima Hospital, Nerima, Tokyo, 117-8521 Japan; Critical Care Medicine, Jikei University School of Medicine, Minato, Tokyo, 105-0003 Japan; Emergency and Critical Care Medicine, National Hospital Organization Disaster Medical Center, Tachikawa, Tokyo, 190-0014 Japan; Department of Intensive Care Medicine, Saiseikai Yokohamashi Tobu Hospital, Yokohama-shi, Kanagawa, 230-8765 Japan; Department of Emergency and Critical Care Medicine, Social Insurance Chukyo Hospital, Nagoya, Aichi, 457-0866 Japan; Emergency Medical Center, Nagasaki University Hospital, Nagasaki, Nagasaki, 852-8102 Japan; Emergency and Critical Care Medical Center, Osaka City General Hospital, Miyakohuma, Osaka, 534-0021 Japan; Intensive Care Unit, Kobe City Medical Center General Hospital, Kobe City, Hyogo, 650-0047 Japan; Department of Anesthesia and Intensive Care, Hiroshima City Hospital, Hiroshima, Hiroshima, 730-8518 Japan; Advanced Medical Emergency and Critical Care Center, Yamaguchi University Hospital, Ube City, Yamaguchi, 755-8505 Japan; Intensive Care Unit, Nagasaki University Hospital, Nagasaki, Nagasaki, 852-8102 Japan; Department of Emergency and Critical Care Medicine, Nippon Medical School Tama Nagayama Hospital, Tama-shi, Tokyo, 206-8512 Japan; Department of Emergency and Critical Care Medicine, Nippon Medical School Chiba Hokusou Hospital, Inzai-shi, Chiba, 270-1694 Japan; Department of Emergency and Critical Care Medicine, Nippon Medical School, Bunkyo-ku, Tokyo, 113-8603 Japan

**Keywords:** Acute pulmonary edema, Acute respiratory distress syndrome, Indirect injury, Direct injury, Pulmonary vascular permeability, Transpulmonary thermodilution technique

## Abstract

**Background:**

Acute respiratory distress syndrome (ARDS) is characterized by the increased pulmonary permeability secondary to diffuse alveolar inflammation and injuries of several origins. Especially, the distinction between a direct (pulmonary injury) and an indirect (extrapulmonary injury) lung injury etiology is gaining more attention as a means of better comprehending the pathophysiology of ARDS. However, there are few reports regarding the quantitative methods distinguishing the degree of pulmonary permeability between ARDS patients due to pulmonary injury and extrapulmonary injury.

**Methods:**

A prospective, observational, multi-institutional study was performed in 23 intensive care units of academic tertiary referral hospitals throughout Japan. During a 2-year period, all consecutive ARDS-diagnosed adult patients requiring mechanical ventilation were collected in which three experts retrospectively determined the pathophysiological mechanisms leading to ARDS. Patients were classified into two groups: patients with ARDS triggered by extrapulmonary injury (ARDSexp) and those caused by pulmonary injury (ARDSp). The degree of pulmonary permeability using the transpulmonary thermodilution technique was obtained during the first three intensive care unit (ICU) days.

**Results:**

In total, 173 patients were assessed including 56 ARDSexp patients and 117 ARDSp patients. Although the Sequential Organ Failure Assessment (SOFA) score was significantly higher in the ARDSexp group than in the ARDSp group, measurements of the pulmonary vascular permeability index (PVPI) were significantly elevated in the ARDSp group on all days: at day 0 (2.9 ± 1.3 of ARDSexp vs. 3.3 ± 1.3 of ARDSp, *p* = .008), at day 1 (2.8 ± 1.5 of ARDSexp vs. 3.2 ± 1.2 of ARDSp, *p* = .01), at day 2 (2.4 ± 1.0 of ARDSexp vs. 2.9 ± 1.3 of ARDSp, *p* = .01). There were no significant differences in mortality at 28 days, mechanical ventilation days, and hospital length of stay between the two groups.

**Conclusions:**

The results of this study suggest the existence of several differences in the increased degree of pulmonary permeability between patients with ARDSexp and ARDSp.

**Trial registration:**

This report is a sub-group analysis of the study registered with UMIN-CTR (IDUMIN000003627).

## Background

Acute respiratory distress syndrome (ARDS) is a challenging disease renowned to any intensive care unit (ICU) and is associated with a high mortality rate, as documented by recent systematic reviews finding a pooled mortality of 44% and 43% [1, 2].

ARDS is characterized by the increased pulmonary capillary permeability secondary to diffuse alveolar inflammation and injuries [3]. Common risk factors can be classified into two groups: extrapulmonary causes (indirect etiologies: ARDSexp) or pulmonary causes (direct etiologies: ARDSp). The major causes of ARDSexp include non-pulmonary sepsis, major trauma, pancreatitis, severe burns, non-cardiogenic shock, drug overdose, and multiple transfusions, while the main causes of ARDSp are pneumonia, aspiration of gastric contents, inhalation injury, pulmonary contusion, pulmonary vasculitis, and drowning [4]. Lung injuries of different origins may hold distinct pathophysiology, lung morphology, radiology, respiratory mechanics, and response to managements [5]. This distinction between an indirect and a direct etiology of lung injury is gaining more attention as a means of better comprehending the pathophysiology of ARDS and possibly for modifying ventilator management [6, 7]. Several studies have reported the difference between respiratory mechanics and severity between ARDSexp and ARDSp [6–8]. Gattinoni et al. [6] measured the elastic properties of the lung and chest wall by using the quasistatic technique and reported that the differences in respiratory mechanics and response to positive end-expiratory pressure (PEEP) between patients with ARDSexp and those with ARDSp were predominantly due to collapse. On the other hand, a systematic review consisting of 34 studies exhibited no difference in mortality between both groups [9]. Presently, we believe that radiological imaging, respiratory mechanics, as well as response to PEEP features between both groups do not affect mortality. We consider that no difference in mortality between both groups may depend on the same therapeutic strategy given to all ARDS cases. ARDS patient outcomes may be improved with specific modified therapies that would address the underlying pathologies during the initial stage. We believe that more quantitative methods estimating the heterogeneity of ARDS at the initial stage and a customized therapy for each individual case may be helpful to appropriately manage ARDS patients.

There are few quantitative methods that distinguish between both ARDS categories during their initial stages. As ARDS is a syndrome and includes various clinical conditions, the absolute difference between ARDSexp and ARDSp is still unknown. We hypothesized that the degree of increased pulmonary permeability caused by several underlying diseases may be different between patients within the two ARDS categories. We set out to determine whether there were any differences between the two groups in terms of the degree of pulmonary edema during the initial ICU stages.

The transpulmonary thermodilution technique system (PiCCO®, Pulsion Medical Systems, Munich, Germany) is able to establish the degree of pulmonary vascular permeability with the pulmonary vascular permeability index (PVPI) [10]. Kirov et al. [11] reported that the value of PVPI was high in a sheep model with ARDS. Furthermore, a clinical study by Monnet et al. [12] showed that PVPI was higher in patients with ARDS than in those with cardiogenic pulmonary edema. These results suggest that PVPI seemed to be useful to estimate the difference in pulmonary vascular permeability between the types of pulmonary edema [13]. We previously reported that PVPI was significantly higher in ARDS patients than in patients who had cardiogenic pulmonary edema despite both having diffuse bilateral infiltrates of the lung and hypoxemia. A PVPI of 2.6–2.85 was proposed as the differential value to distinguish ‘real’ ARDS with permeability edema from cardiogenic edema or atelectasis which could lead to clinical symptoms that mimic ARDS [14]. Therefore, PVPI might reflect the variety of difference of causes that lead to ARDS.

To our knowledge, little data regarding the difference of PVPI elevation between ARDSexp and ARDSp in the ICU setting is available. The aim of this study was to evaluate the difference in pulmonary vascular permeability, using the value of PVPI between patients with ARDSexp and those with ARDSp.

## Methods

We undertook a prospective, observational, multi-institutional study from March 2009 to August 2011. Patients admitted to the ICU of 23 hospitals in different regions of Japan were screened for ARDS as a sub-analysis of a clinical observational study utilizing hemodynamic monitoring with PiCCO® [14]. The study was approved by the institutional ethics committee, and written informed consent was obtained from all study participants. This study was also registered with the University Hospital Medical Information Network (UMIN) Clinical Trials Registry (UMIN-CTR ID UMIN000003627).

We assessed medical and surgical patients according to the following five criteria: (1) older than 15 years of age (no upper age limit), (2) acute respiratory insufficiency expected to require mechanical ventilatory support in ICU for at least 48 h, (3) PaO_2_/FiO_2_ (P/F) ratio of ≤300 mmHg, (4) bilateral infiltrates on chest radiograph, (5) monitoring by the transpulmonary thermodilution technique as per the discretion of attending physicians. Patients were excluded if one of the following criteria was present: definition of ARDS over 5 days prior to eligibility; past history of chronic respiratory insufficiency (mainly, chronic obstructive pulmonary disease); post lung resection/pneumonectomy; pulmonary thromboembolism; severe peripheral arterial disease; cardiogenic shock (cardiac index <1.5 L/min/m^2^); acute phase of trauma with lung contusion, drowning, and burns; unsuitable situation for monitoring with PiCCO®. Three or more experts (intensive care, respiratory, and cardiology) determined the diagnosis of ARDS taking into account medical history, clinical features involving computed tomography (CT) appearances, respiratory and hemodynamic variables, and clinical course with therapy. We excluded all cases of respiratory failure that were caused by massive pneumothorax, atelectasis, and pleural effusion. Any case with an extravascular lung water index (EVLWI) <10 mL/kg was excluded [14].

The following data were collected from the medical records: age, gender, underlying disease, hospital length of stay, length of mechanical ventilation, and clinical outcome at 28 days (survival or death). We also calculated the Acute Physiology and Chronic Health Evaluation (APACHE) II score, Sequential Organ Failure Assessment (SOFA) score, PEEP, and P/F ratio at day 0. Once the above-mentioned criteria were fulfilled, we commenced PiCCO® measurement data collection in which all patients were monitored for three consecutive days: day 0, day 1, and day 2. PVPI and extravascular lung water (EVLW) were measured concurrently. At the same time, intrathoracic blood volume (ITBV) was measured as volumetric parameters by PiCCO®. We calculated EVLWI with predicted body weight and intrathoracic blood volume index (ITBI) with area of body surface. We utilized predicted body weight to index EVLW and ITBV according to previous studies which determined that EVLWI indexed to the predictive body weight was a better prognostic indicator for ARDS patients than EVLWI indexed to the actual body weight [15, 16].

From the various diagnosed causes of ARDS, we classified all patients into two groups (indirect injury group or direct injury group). We assessed each patient's medical history, clinical presentation, chest CT, radiography, and echocardiography. Indirect injury group (ARDSexp) included patients with ARDS secondary to non-pulmonary sepsis, major trauma, pancreatitis, severe burns, non-cardiogenic shock, drug overdose, and multiple transfusions. The direct injury group (ARDSp) included patients with ARDS caused by pneumonia, aspiration of gastric contents, inhalation injury, pulmonary contusion, pulmonary vasculitis, and drowning [4]. Patients that presented with acute phase of inhalation burn, pulmonary contusion, and drowning were excluded from the study. All the experts who conducted the classification were blinded to the value of PVPI.

### Statistical analysis

Descriptive frequencies were expressed using the mean and standard deviation (SD). Categorical data was analyzed using Pearson's chi-square test for the characteristics and 28-day mortality. The independent *t* test was used for normally distributed variables, or the Mann–Whitney *U* test was used for non-normally distributed variables. All tests were two-sided, and *p* < .05 was considered to be significant. We analyzed all data using SPSS software, version 18 for Windows (SPSS, Chicago, IL, USA).

## Results

**Figure 1:**
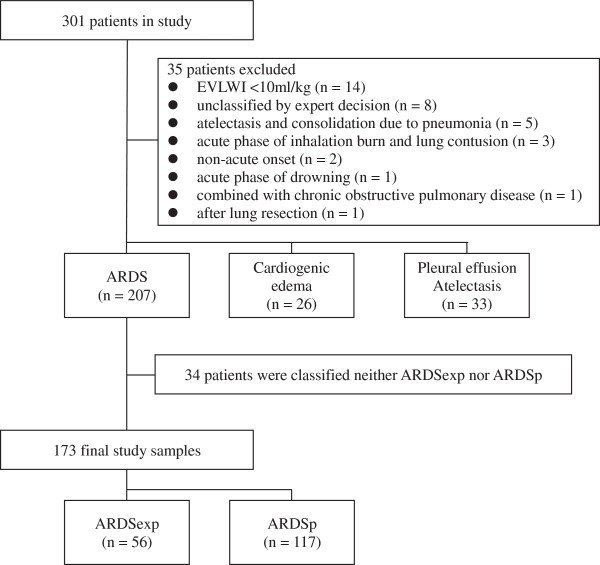
**Patient flow diagram.**

During the study period from March 2009 to August 2011, we assessed a total of 301 patients that were subjected to mechanical ventilation. Of these, 94 were excluded due to reasons presented in Figure 1. Of the remaining 207 cases with absolute ARDS, the experts categorized 34 patients as neither ARDSexp nor ARDSp groups due to lack of a clear identification of the underlying cause or a mixed clinical presentation of the disease. The remaining 173 patients were included into the final analysis.

Patients' characteristics are summarized in Table 1. Of these, the age range of affected patients was 21–93 years (median 67 years), with 67% males. Fifty-six patients (32%) had a diagnosis of ARDSexp, and 117 patients (68%) had a diagnosis of ARDSp. The most common cause of ARDSexp was sepsis (71%) and pneumonia (80%) in ARDSp. Significant differences in baseline characteristics were seen between the two groups with the SOFA score (ARDSexp 12 ± 4 vs. ARDSp 10 ± 3; *p* = .0001) and the concentration of serum albumin (2.4 g/dL of ARDSexp vs. 2.7 g/dL of ARDSp; *p* = .02). There were no significant differences in APACHE II, PEEP, and mean airway pressure initially. The P/F ratio tended to be lower in ARDSp than ARDSexp (163 ± 73 of ARDSexp vs. 143 ± 70 of ARDSp; *p* = .09).

**Table 1:** **Characteristics of 173 patients at the initial measurement of thermodilution technique**

	ARDSexp	ARDSp	***p*** value
	***n*** = 56	***n*** = 117	
Age (year), median	65	72	.03
Male, *n* (%)	32 (57)	84 (72)	.05
Causes of ARDS, *n* (%)			
Sepsis	40 (71)		
Trauma and burn	10 (18)		
Pancreatitis	4 (7)		
TRALI	2 (4)		
Pneumonia		94 (80)	
Aspiration		20 (17)	
Lung contusion		2 (2)	
Inhalation burn		1 (1)	
APACHE II score	24 ± 9	23 ± 8	.39
SOFA score	12 ± 4	10 ± 3	.0001
PEEP (cmH_2_O)	9 ± 5	9 ± 5	.36
PaO_2_/FiO_2_ ratio	163 ± 73	143 ± 70	.09
Mean airway pressure (cmH_2_O)	17 ± 6	17 ± 4	.55
Na (mmol/L)	135 ± 19	138 ± 7	.37
K (mmol/L)	4.1 ± 0.8	4.0 ± 0.9	.15
Albumin (g/dL)	2.4 ± 0.5	2.6 ± 0.8	.02
Glucose (mg/dL)	159 ± 61	165 ± 83	.95
Blood urea nitrogen (mg/dL)	38 ± 28	36 ± 25	.84

Values are given as mean ± standard deviation, unless otherwise indicated. *ARDS* acute respiratory distress syndrome, *ARDSexp* acute respiratory distress syndrome secondary to extrapulmonary cause, *ARDSp* acute respiratory distress syndrome secondary to pulmonary cause, *TRALI* transfusion-related acute lung injury, *APACHE* Acute Physiologic and Chronic Health Evaluation, *SOFA* Sequential Organ Failure Assessment, *PEEP* positive end-expiratory pressure.

**Figure 2:**
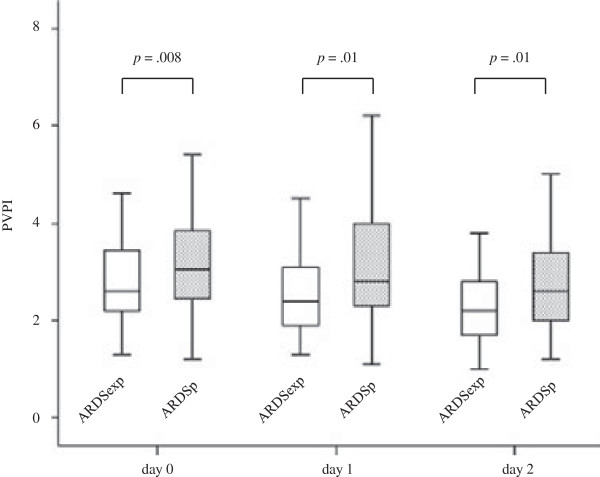
**Comparison of PVPI between ARDSexp and ARDSp during the study period.** The measurement of PVPI was significantly elevated in ARDSp compared with ARDSexp on all days: at day 0 (2.9 ± 1.3 of ARDSexp vs. 3.3 ± 1.3 of ARDSp, *p* = .008), at day 1 (2.8 ± 1.5 of ARDSexp vs. 3.2 ± 1.2 of ARDSp, *p* = .01), at day 2 (2.4 ± 1.0 of ARDSexp vs. 2.9 ± 1.3 of ARDSp, *p* = .01).

Results of measurements of PiCCO® at day 0, day 1, and day 2 are summarized in Table 2. The measurement of PVPI (Figure 2) was significantly elevated in ARDSp compared with ARDSexp on all days: at day 0 (2.9 ± 1.3 of ARDSexp vs. 3.3 ± 1.3 of ARDSp, *p* = .008), at day 1 (2.8 ± 1.5 of ARDSexp vs. 3.2 ± 1.2 of ARDSp, *p* = .01), at day 2 (2.4 ± 1.0 of ARDSexp vs. 2.9 ± 1.3 of ARDSp, *p* = .01). All ITBI data from both groups was in the upper range of normal. Although there was no significant difference in the EVLWI at day 0 and day 1, the level of EVLWI in the ARDSp group was significantly higher than that in the ARDSexp patients at day 2 (14.9 ± 6.0 of ARDSexp vs. 17.6 ± 7.8 of ARDSp, *p* = .02).

**Table 2:** **Results of the measurement by thermodilution technique**

	ARDSexp	ARDSp	***p***value
	***n*** = 56	***n*** = 117	
Day 0			
ITBI	1,067 ± 236	1,015 ± 270	.09
EVLWI	17.8 ± 6.6	19.0 ± 7.1	.20
PVPI	2.9 ± 1.3	3.3 ± 1.3	.008
Day 1			
ITBI	1,069 ± 309	1,019 ± 279	.37
EVLWI	17.0 ± 8.0	18.0 ± 7.0	.16
PVPI	2.8 ± 1.5	3.2 ± 1.2	.01
Day 2			
ITBI	1,095 ± 263	1,078 ± 313	.41
EVLWI	14.9 ± 6.0	17.6 ± 7.8	.02
PVPI	2.4 ± 1.0	2.9 ± 1.3	.01

Values are given as mean ± standard deviation. *ARDSexp* acute respiratory distress syndrome secondary to extrapulmonary cause, *ARDSp* acute respiratory distress syndrome secondary to pulmonary cause, *ITBI* intrathoracic blood volume index, *EVLWI* extravascular lung water index, *PVPI* pulmonary vascular permeability index.

Patients' outcomes are summarized in Table 3. There was no significant difference in 28-day mortality, hospital stay days, and ventilation days between the two groups as follows: mechanical ventilation (16 ± 19 days of ARDSexp vs. 13 ± 9 days of ARDSp, *p* = .85), hospital length of stay (42 ± 55 days of ARDSexp vs. 40 ± 56 days of ARDSp, *p* = .51), and mortality at 28 days (32% of ARDSexp vs. 44% of ARDSp, *p* = .15).

**Table 3:** **Clinical outcomes for participants**

	ARDSexp	ARDSp	***p*** value
	***n*** = 56	***n*** = 117	
28-day mortality, *n* (%)	18 (32)	51 (44)	.15
Hospital stay days	42 ± 55	40 ± 56	.51
Ventilation days	16 ± 19	13 ± 9	.85

Values are given as mean ± standard deviation, unless otherwise indicated.

## Discussion

ARDS is considered to be an expression of a diffuse inflammatory reaction within the lungs caused by a number of underlying diseases, which have a variety of pathophysiological courses being either of pulmonary or extrapulmonary origin. Thus, depending on its etiology, ARDS may present with obvious distinct characteristics. In this study, we attempted to establish the quantitative difference in pulmonary permeability between ARDSexp and ARDSp with PVPI obtained by the transpulmonary thermodilution technique (PiCCO®).

The characteristics and clinical outcomes in both groups were similar to previous studies [6, 8, 17, 18]. The prevalence of ARDSp in this study was higher compared to that of the ARDSexp group which mainly consisted of patients with sepsis and trauma, as opposed to ARDSp which consisted of those with pneumonia and aspiration [6, 8, 17, 18]. Although these various clinical backgrounds may influence ARDS outcomes, no significant differences in 28-day mortality, length of hospital stay, and number of mechanical ventilation days were noted between the ARDSexp or ARDSp patients in this study. These results concur with a previously reported meta-analysis which manifested no difference in mortality between ARDSexp and ARDSp [9]. Agarwal et al. [8] also reported that categories of underlying disease did not affect the duration of hospitalization and the number of mechanical ventilation days, although this study showed that the initial severity of respiratory failure, PaO_2_/FiO_2_ ratio, was worse in patients with ARDSp than in those with ARDSexp. Each of the two ARDS groups characterized by different origins arguably has a different pathophysiological course for the lung permeability along with similar clinical syndromes and outcomes. Hence, the initial separation of ARDSexp and ARDSp appears to be futile for predicting outcome from ARDS.

The value of ITBI as volumetric parameters by PiCCO® in both groups was in the upper normal range. All participants were managed with appropriate fluid therapy during their admission to ICU. On the other hand, the value of PVPI was extremely elevated above the normal range (PVPI of 2.6–2.85; differential value to diagnose real ARDS [14]). We believe that these results suggest that all the included cases demonstrated permeability pulmonary edema, namely real ARDS with no cases of cardiogenic pulmonary edema or fluid overload which could be one of the most important biases to assess permeability pulmonary edema.

Patients with ARDSexp had significantly higher SOFA scores and lower albumin concentrations similarly to the report by Agarwal et al. [8]. We found PVPI in the ARDSexp group to be lower than that in those with ARDSp and speculate that the direct insult (i.e., caused by pulmonary infection, aspiration, and trauma) may have influenced the pulmonary permeability to a greater extent than the indirect insult with systematic hyper-cytokine storm caused by sepsis. We also noted EVLWI in the ARDSexp group to be lower than that in the ARDSp group. Generally, lower albumin concentrations lead to lower osmotic pressure, so that patients with hypoalbuminemia suffer from whole body edema, ascites, and especially pericardial effusion. Nevertheless, we found that direct insult caused the increase of EVLWI greater than systematic inflammation and hypoalbuminemia. This result could correspond to the animal report which compared the response of pulmonary epithelium damage caused by intraperitoneally or intratracheally exposed lipopolysaccharide and which concluded that the intratracheal insult leads to greater lung inflammation and ultrastructural morphologic changes. The levels of inflammatory cytokines (interleukin-6, interleukin-8 function homolog, and interleukin-10) found in the bronchoalveolar lavage fluid were significantly elevated in pulmonary ARDS models compared to extrapulmonary ARDS models, whereas no differences were observed in the number of infiltrating neutrophils [19]. This suggests, therefore, that the level of inflammation and the activation of neutrophils in patients with ARDSp may be higher than those in patients with ARDSexp.

There are several limitations to this study. First, the sample size was relatively small due to strict exclusion criteria to omit cases with complications that might also lead to hypoxemia and bilateral infiltrate on chest X-ray, including massive pneumothorax, atelectasis, and pleural effusion, to ensure that we included real ARDS patients. Second, the numbers of ARDSexp were fewer than those of ARDSp (56 of ARDSexp vs. 117 of ARDSp). There might be some statistical bias. We also excluded 94 cases with acute respiratory failure resulting from cardiogenic pulmonary edema, plural effusion, and atelectasis that might resemble ARDS (i.e., hypoxia with diffuse bilateral infiltrates) despite the lack of increased pulmonary permeability. We are concerned that our strict exclusion criteria may have excluded those with severe respiratory failure, and participants were limited to more moderate cases. However, we believe that our exclusion criteria provided an appropriate estimate of the key features of ARDS, namely participants with increased pulmonary permeability edema. We also excluded cases suspected of having both etiologies (ARDSexp and ARDSp) from our analysis. Finally, the varied and complicated factors of the underlying disease might influence the accuracy of thermodilution technique measurements. For this matter, we did not compare all those factors that could affect the accuracy of PiCCO® findings but rather only the cases that were deemed possible of adequate PiCCO® monitoring.

To our knowledge, there are few reports considering quantitative methods distinguishing both types of ARDS within the ICU setting. In a clinical situation, timely recognition of the difference and course in an ARDS lung is essential to drive and inform the clinical decision processes and dictate specific therapeutic strategies. The quantitative differences in EVLWI and PVPI obtained by the PiCCO® system may be one important index that indicates a pathological difference between ARDSexp and ARDSp by estimating the amount of pulmonary permeability. Monitoring with PiCCO® might give us timely quantitative information to make more informed clinical decisions.

## Conclusions

The results of this study suggest that the PiCCO® monitoring could be useful to distinguish between the state of pulmonary permeability in ARDSexp and ARDSp patients. We believe that there is a fundamental difference in the pathophysiology between these ARDSexp and ARDSp groups. Utilizing PVPI may provide us with timely quantitative information of pulmonary permeability in these two forms of ARDS. Additional studies should be undertaken in order to determine whether the clinical strategy guided with PVPI can have an impact on outcome and improve hypoxemia for patients with ARDS.

## Abbreviations

APACHE II: Acute Physiology and Chronic Health Evaluation II
ARDS: acute respiratory distress syndrome
ARDSexp: ARDS triggered by extrapulmonary injury
ARDSp: ARDS triggered by pulmonary injury
CT: computed tomography
EVLW: extravascular lung water
EVLWI: extravascular lung water index
ICU: intensive care unit
ITBI: intrathoracic blood volume index
P/F ratio: PaO_2_/FiO_2_ ratio
PEEP: positive end-expiratory pressure
PVPI: pulmonary vascular permeability index
SOFA: Sequential Organ Failure Assessment.
